# Factors associated with inappropriate antibiotic use in South African communities: findings from the CAMUS survey

**DOI:** 10.3389/fphar.2026.1771309

**Published:** 2026-03-20

**Authors:** Nishana Ramdas, Natalie Schellack, Corrie Uys, Brian Godman, Stephen M. Campbell, Johanna C. Meyer

**Affiliations:** 1 Department of Public Health Pharmacy and Management, School of Pharmacy, Sefako Makgatho Health Sciences University, Ga-Rankuwa, South Africa; 2 Department of Pharmacology, Faculty of Health Sciences, University of Pretoria, Pretoria, South Africa; 3 Applied Microbial and Health Biotechnology Institute (AMHBI), Cape Peninsula University of Technology, Cape Town, South Africa; 4 Strathclyde Institute of Pharmacy and Biomedical Sciences, University of Strathclyde, Glasgow, United Kingdom; 5 Antibiotic Policy Group, City St. George’s, University of London, London, United Kingdom; 6 School of Health Sciences, University of Manchester, Manchester, United Kingdom; 7 South African Vaccination and Immunisation Centre, Sefako Makgatho Health Sciences University, Ga-Rankuwa, South Africa

**Keywords:** antimicrobial resistance, CAMUS, health literacy, patient behaviors, patient expectations, primary healthcare, social norms, South Africa

## Abstract

**Background:**

Inappropriate antibiotic use in the community is a primary driver of antimicrobial resistance (AMR), particularly in low- and middle-income countries (LMICs) where ambulatory care, including primary healthcare (PHC) facilities, is the main point of contact. While stewardship efforts in South Africa have focused largely on prescribers and hospitals, the behavioral drivers of community antimicrobial use, specifically the *‘demand side’*, remain poorly understood. This study aimed to address this gap by applying the newly developed Community Antimicrobial Use Scale (CAMUS) to quantify the prevalence of misuse and identify potential independent sociodemographic and behavioral factors associated with inappropriate antibiotic use among ambulatory care patients in South Africa.

**Methods:**

A cross-sectional survey was conducted among 1,283 adult patients attending 25 ambulatory care public-sector facilities in two provinces. Data was collected using the Community Antimicrobial Use Scale (CAMUS) and the Health Literacy Test for Limited Literacy Populations (HELT-LL). We quantified patterns of knowledge, social norms, expectations, and non-prescribed use. Multivariable logistic regression models examined determinants of misuse and antibiotic demand.

**Results:**

General awareness of antibiotics existed, but major misconceptions persisted regarding their role in viral infections. Notably, 76.5% of participants attending public-sector facilities believed antibiotics can treat colds and influenza. Non-prescribed use was reported by nearly a quarter of participants, with community pharmacies identified as the primary source (81.7%). While factual knowledge gaps were present, multivariable models revealed that social norms favoring non-prescribed use and inadequate health literacy were the strongest predictors of misuse. Patients with inadequate health literacy were significantly more likely to try and obtain antibiotics without a prescription compared to those with adequate literacy (aOR=13.86; 95% CI: 2.60–73.93).

**Conclusion:**

In this large sample of ambulatory care public-sector patients, inappropriate antibiotic use appeared to be linked with permissive social norms and health literacy gaps rather than knowledge deficits alone. Interventions that focus solely on correcting factual misconceptions may be insufficient to address inappropriate antibiotic use. Future stewardship strategies must explicitly target social norms, family influences, and pharmacy behavior, alongside strengthening patient-prescriber-pharmacy communication.

## 1 Introduction

Antimicrobial resistance (AMR) is as a major global health threat, responsible for an estimated 1.27 million deaths directly attributable to drug-resistant bacterial infections in 2019 (Murray et al., 2022; Patra et al., 2025; Reza et al., 2025). AMR is also associated with appreciable costs (Siaba et al., 2025; Totaro et al., 2025). The burden of AMR is highest among low- and middle-income countries (LMICs), including among African countries, where infectious diseases remain prevalent and health systems face appreciable resource constraints (Antimicrobial Resistance Collaborators, 2024; Lubanga et al., 2024; Sartorius et al., 2024). AMR will continue to increase with the potential for up to 4.1 million AMR-related deaths each year in sub-Saharan Africa alone by 2050 without additional actions (Totaro et al., 2025). In South Africa, national surveillance data also demonstrate resistance to key pathogens including *Acinetobacter baumannii*, *Escherichia coli*, *Klebsiella pneumoniae*, and *Staphylococcus aureus* including methicillin-resistant *Staphylococcus aureus*. Current resistance rates have prompted concerns with the adequacy of national responses (Finlayson et al., 2024; National Department of Health, Republic of South Africa, 2024; Mendelson et al., 2025). Recent activities in South Africa include calls to the Minister of Health to re-invest time and resources to update the National Action Plan on AMR to address rising AMR (Mendelson et al., 2022; Mendelson et al., 2025).

A key contributor to AMR is the inappropriate use of antibiotics in the community. This includes unnecessary prescribing for self-limiting viral infections such as upper respiratory tract infections (URTIs), high levels of dispensing of antibiotics without a prescription including for URTIs, storage and use of leftover antibiotics, as well as early discontinuation of treatment once patients feel better due to resource issues (Li et al., 2023; Abejew et al., 2024; Sono et al., 2024a; Saleem et al., 2025a; Saleem et al., 2025b; Song et al., 2025). For the purposes of this study, non-prescribed antibiotic use is defined as antibiotics dispensed from community pharmacies without a prescription, antibiotics sourced from family members or friends without consulting a healthcare professional (HCP), or the use of leftover antibiotics from previous incomplete courses (Anstey Watkins et al., 2019; Saleem et al., 2025a). Ambulatory care is the critical setting for addressing AMR in LMICs with antibiotic utilization in this sector accounting for up to 95% of total antibiotic use in humans (Duffy et al., 2018). While hospital-based antimicrobial stewardship (AMS) activities have expanded in recent years across Africa, including South Africa, the *“demand-side”* determinants, patients’ expectations, social norms, health literacy, and their impact on AMR, especially in primary care, have received less attention (Otieno et al., 2022; Siachalinga, et al., 2022; Chigome et al., 2025a; Ramdas et al., 2025a). However, this is beginning to change with an increasing number of antimicrobial stewardship programmes (ASPs) now being undertaken in the community among LMICs, which include African countries (De Vries et al., 2022; van Hecke et al., 2024; Chigome et al., 2025a; Saleem et al., 2025a).

Evidence from South Africa suggests that clinicians in both public and private primary care facilities frequently report patient pressure, whether actual or perceived, as a driver of unnecessary antibiotic prescribing (Farley et al., 2018; De Vries et al., 2022; Guma et al., 2022; Lagarde and Blaauw, 2023). Such pressures exacerbate current sub-optimal prescribing of antibiotics in primary care in South Africa (Gasson et al., 2018; Manderson, 2020; Truter and Knoesen, 2018; Chigome et al., 2023; Chigome et al., 2025a; Chigome et al., 2025b; Maluleke et al., 2025a; Maluleke et al., 2026a). However, most existing studies on patient perspectives rely on *ad hoc* knowledge, attitudes, and practice (KAP) surveys that typically lack theoretical grounding (McCullough et al., 2016; Auta et al., 2019; Waaseth et al., 2019; Ramdas et al., 2025a).

To address this gap, this study utilized the newly validated Community Antimicrobial Use Scale (CAMUS) (Ramdas et al., 2025b) to firstly quantify the prevalence of antibiotic misconceptions, social norms, and non-prescribed use among adult attendees at ambulatory care public-sector facilities; and secondly, identify the independent sociodemographic and behavioral factors associated with inappropriate antibiotic use. By moving beyond descriptive surveillance, these findings are intended to inform targeted interventions to improve AMS activities in ambulatory care, supporting the further development of South Africa’s National Action Plan on AMR with robust, theory-driven evidence.

## 2 Materials and methods

### 2.1 Study design and setting

This cross-sectional analytical study was conducted across 25 public healthcare facilities in South Africa. To capture the necessary geographic and socioeconomic diversity while maintaining operational feasibility, a multi-stage pragmatic sampling strategy was employed. First, study districts were identified based on the logistical footprint of the trained field data collection teams. This approach ensured the inclusion of two distinct demographic profiles, that is Limpopo province (representing deep rural/semi-rural populations) and Gauteng province (representing high-density urban/peri-urban populations). Second, to minimize selection bias within these districts, individual ambulatory care public-sector facilities were randomly selected from the provincial facility lists. Ambulatory care public-sector facilities were selected for the study setting because they serve approximately 84% of the South African population and constitute the primary entry point into the healthcare system (Meyer et al., 2017; National Department of Health, Republic of South Africa, 2024). This setting ensured the assessment of antibiotic demand and expectations within the tier where national stewardship policies are primarily implemented.

Ambulatory care public-sector facilities were chosen for this study since at the time of the study published studies had shown very variable rates of dispensing of antibiotics without a prescription in South Africa (Anstey Watkins et al., 2019; Do et al., 2021; Mokwele et al., 2022; Sono et al., 2024b). Where antibiotics were being dispensed without a prescription, this was typically for patients with urinary tract infections (UTIs), or UTIs along with sexually transmitted infections (STIs), rather than for patients with URTIs (Mokwele et al., 2022; Sono et al., 2024a).

### 2.2 Theoretical framework and instrument development

Unlike traditional knowledge, attitude, and practice (KAP) surveys, which are often atheoretical, the CAMUS instrument was developed using a composite behavioral framework to capture the complex psychosocial drivers of antibiotic use. The instrument constructs were explicitly mapped to three established behavioral theories.

1. The Health Belief Model (HBM): To measure patients’ *perceived susceptibility* to illness and *perceived severity* of symptoms (e.g., the belief that antibiotics are necessary to prevent a cold from becoming serious) (Rosenstock, 1974).
2. The Theory of Planned Behavior (TPB): To assess *subjective norms* (social pressure from family/community) and *perceived behavioral control* (Ajzen, 1991).
3. Social Cognitive Theory (SCT): To evaluate *outcome expectations* (the belief that antibiotics cure viral infections) and *observational learning* (modelling behavior based on community habits) (Bandura, 1986).

The instrument underwent rigorous psychometric evaluation to ensure validity and reliability. A pilot study was conducted with 30 participants to assess feasibility and face validity, informing minor linguistic adaptations. Construct validity was established through Factor Analysis, and internal consistency with Cronbach’s alpha coefficients ranging from 0.72 to 0.90 for the five domains (Ramdas et al., 2025b).

### 2.3 Study participants

A convenience sample of 1,283 adult patients (≥18 years) was recruited from the waiting areas of the selected ambulatory care public-sector facilities. This was seen as the most productive source for potential participants, since, as mentioned, at the time there was conflicting findings regarding the extent of dispensing of antibiotics without a prescription in South Africa. To structure the recruitment process, trained independent field workers approached eligible patients consecutively as they waited in line for their consultations. To ensure that participation did not interfere with or delay urgent care, field workers were instructed to approach only those patients who appeared observationally stable. To help with this process, all independent field workers were qualified pharmacists (Master of Pharmacy students) possessing clinical knowledge. However, “medical stability” was not determined through a formal facility triage or diagnostic screening tool. Instead, this operational procedure relied on their professional observational judgement to purposefully exclude individuals in overt, acute physical distress, e.g., those appearing to experience severe pain, shortness of breath, actively vomiting, or requiring immediate emergency attention. This was because we felt we could not be overly intrusive in the waiting area by asking each patient their medical history before asking for their help with the questionnaire, especially those in obvious distress. In addition, an ethical requirement was that this research did not delay urgent medical care. Unfortunately, data on the exact number of patients who refused participation were not systematically recorded during the fieldwork as the focus was on recruiting as many patients as possible within a reasonably short time scale. This means that a formal response rate cannot be calculated.

Participation was subsequently restricted to those patients who were willing to provide informed consent as well as capable of communicating in English, as the CAMUS instrument at the time was validated in this language only to ensure measurement validity (Ramdas et al., 2025b). We acknowledge that this inclusion criterion likely resulted in a study population with a slightly higher educational profile than the general public, as functional English proficiency in South Africa is strongly correlated with formal schooling (Posel and Zeller, 2016).

We explicitly acknowledge that our reliance on a convenience sampling approach, combined with the lack of refusal data, introduces inherent selection bias. It is possible that individuals who agreed to participate may possess different health-seeking behaviors, literacy levels, or social attitudes compared to those who declined or were not approached.

Despite this inherent bias, the sample size was robust for the planned analyses. The target sample size was calculated *a priori* to ensure adequate statistical power across the three distinct data collection sites. Using Epi Info™ software across the respective cohorts, assuming a conservative expected frequency of 50%, a 5% margin of error, and a 95% confidence level against the respective district population estimates, the minimum required sample sizes were calculated as 383, 381 (prospectively adjusted to 420 to account for incomplete data), and 371 (adjusted to 408). This yielded a cumulative minimum target of 1,211 participants. To maximize statistical power, all-inclusive convenience sampling was employed until these targets were met, resulting in a final recruited sample of 1,283, which successfully exceeded the *a priori* calculation. Furthermore, this final sample size substantially surpasses established methodological benchmarks for psychometric validation, which recommend a minimum participant-to-item ratio of 10:1 for robust factor analysis (Boateng et al., 2018); for the 30-item CAMUS, our ratio was highly robust at 42:1. Finally, the sample size also satisfied established guidelines for regression modelling. Based on the formula n = 100 + 50i (where i represents the number of independent variables) recommended for large observational studies (Bujang et al., 2018), our generalized linear model containing 13 independent variables required a minimum of 750 participants, a threshold our sample of 1,283 comfortably exceeded.

### 2.4 Data collection

Data were collected via face-to-face interviews using a digital version of the CAMUS instrument hosted on Google Forms. Trained field workers administered the survey using electronic tablets to record responses. The survey instrument (CAMUS) comprised three sections.

1. Sociodemographic characteristics: Age, sex, education, employment status, residential setting, and province.
2. The CAMUS instrument: Assessing the five behavioral domains (statistically identified as Factors 1–5) comprising: Understanding of antibiotics; Social/behavioral norms; Non-prescribed use; Understanding of AMR; and Attitudes/beliefs.
3. Health literacy assessment: To evaluate the relationship between literacy and antibiotic use, the Health Literacy Test for Limited Literacy Populations (HELT-LL) (Marimwe and Dowse, 2019) was administered.
4. Due to the significant time burden of this assessment and logistical constraints, the tool was administered to a nested sub-sample of participants (n=463) primarily within the Gauteng study sites. To ensure this pragmatic selection did not introduce demographic bias, a sensitivity analysis compared the HELT-LL participants (n=463) against the non-selected group (n=813). Chi-square analyses revealed statistically significant differences regarding age (p < 0.001), gender (p < 0.001), and employment status (p < 0.001), with the sub-sample being generally older and more likely to be employed (see Supplementary Table S1). To mitigate the impact of these disparities, all subsequent multivariable models were adjusted for age, sex, and employment status.

### 2.5 Data management and statistical analysis

Data were exported to Microsoft Excel for cleaning and analyzed using IBM SPSS Statistics (Version 30.0). The analysis followed a three-stage approach, outlined in Box 1.

BOX 1 **– Three-staged approach to data analysis.**

•Descriptive statistics: Frequencies and percentages were calculated to summarize participant sociodemographic and item responses across the five CAMUS domains.
•Bivariate analysis: Associations between antibiotic misuse behaviors and sociodemographic or contextual variables were assessed using Pearson’s Chi-square tests. Where cell counts were sparse (expected count <5), Monte Carlo significance testing was employed to obtain robust p-values.
•Multivariable analysis: Generalized linear models (GLM) were fitted to identify factors associated with non-prescribed use. A binomial distribution (logit link) was used for binary outcomes, and a Gamma distribution (log link) was used for continuous factor scores to account for the positively skewed distribution of the misuse behavior data.

Model diagnostics, including goodness-of-fit statistics (AIC, BIC, and Omnibus Likelihood Ratio tests), were performed to ensure model stability (see Supplementary Table S2). All models were adjusted for age, sex, education, employment, residence, and health literacy. Results are reported as adjusted odds ratios (aOR) with 95% confidence intervals (CIs).

### 2.6 Ethical considerations

Ethical approval for this study was obtained from the Sefako Makgatho University Research Ethics Committee (SMUREC/P/220/2023). In addition, approval to conduct the study in both Gauteng and Limpopo provinces was granted by the respective provincial and district research committees. All patient responses were treated with strict confidentiality, and data was securely stored in a password-protected database to ensure compliance with ethical standards and data protection protocols.

To mitigate potential power dynamics and coercion, often inherent in clinical settings, recruitment was conducted by independent field workers distinct from the clinical staff, with no facility personnel involved in the consent process. Patients were explicitly informed that their participation was voluntary, anonymous, and would have absolutely no impact on the care they received at the facility that day or in the future. Informed consent documents explained verbally to all participants prior to signature. This ensured that patients with limited reading proficiency could engage fully with the study requirements without pressure or misunderstanding.

Access to the data was restricted to authorized research personnel only. In addition, no personal identifiable information was included in reports or publications to safeguard participant confidentiality. Data will be stored for a period of 5 years, after all the study results have been published, after which it will be safely destroyed according to institutional policies.

## 3 Results

### 3.1 Participant characteristics

A total of 1,283 participants completed the survey (Table 1). The cohort was predominantly female (60.1%) and African (89.9%), reflecting the typical demographic profile of attendees at South African ambulatory care public-sector facilities. The population was relatively young, with over 60% of participants aged between 18 and 39 years. Socioeconomic vulnerability was evident, with over one-third of the sample (35.7%) reporting unemployment. Educational attainment was varied; while nearly half had completed high school, only a minority (34.9%) held a tertiary qualification. Geographically, the sample captured a diverse range of living conditions, with nearly half of the participants residing in high-density peri-urban townships (46.5%) and a substantial proportion from rural areas (30.2%).

**TABLE 1:** Sociodemographic characteristics of the study participants (N=1,283).

Characteristic	Category	n (%)
Personal demographics		
Sex	Female	771 (60.1)
	Male	505 (39.4)
	Prefer not to disclose	7 (0.5)
Age group (years)	18–29	481 (37.5)
	30–39	309 (24.1)
	40–49	195 (15.2)
	50–59	148 (11.5)
	≥60	150 (11.7)
Ethnicity	African	1,153 (89.9)
	White	76 (5.9)
	Other (coloured, Indian/Asian)	54 (4.2)
Home language	Northern Sotho	460 (35.9)
	Setswana	169 (13.2)
	Zulu	149 (11.6)
	Other languages	505 (39.3)
Socioeconomic status		
Education level	No formal education	33 (2.6)
	Primary school	198 (15.4)
	High school	589 (45.9)
	Tertiary (college/university)	448 (34.9)
	Other	15 (1.2)
Employment status	Employed	532 (41.5)
	Unemployed	458 (35.7)
	Self-employed	151 (11.8)
	Retired/pensioner	142 (11.1)
Geographic context		
Province	Gauteng	876 (68.3)
	Limpopo	401 (31.3)
	Other	6 (0.5)
Residential setting*	Township	596 (46.5)
	Rural area	388 (30.2)
	Suburb	127 (9.9)
	Town	120 (9.4)
	City	52 (4.1)

*Definitions of residential settings: Township: Peri-urban settlements often characterized by high population density and varying infrastructure quality; Rural area: Low-density areas often under traditional authority governance; Suburb: Formal residential neighborhoods located on the periphery of city centers; Town: Smaller semi-urban centers that serve surrounding rural communities; City: High-density urban business and residential cores.

### 3.2 Misconceptions regarding antibiotics and resistance

While participants generally recognized antibiotics as “strong medicines” with potential side effects, specific misconceptions regarding their efficacy for viral infections were widespread (Table 2). A substantial majority (76.5%) incorrectly believed antibiotics can cure common colds and coughs, and over 60% believed antibiotics are effective against viruses. These findings indicate a persistent confusion between viral and bacterial etiologies. Furthermore, general awareness of AMR was low; less than half of the participants (42.2%) could correctly identify the definition of resistance, suggesting that while the term “antibiotic” is familiar, the consequences of its overuse are not well understood.

**TABLE 2:** Participants’ knowledge and misconceptions regarding antibiotics (N=1,283).

Items		Participants’ responses; n (%)		
Description	Correct response	Correct	Incorrect	Don’t know
Antibiotics can cure common colds and coughs	FALSE	204 (15.9)	**982 (76.5)**	97 (7.6)
Antibiotics kill viruses	FALSE	344 (26.8)	**771 (60.1)**	168 (13.1)
Antibiotics can treat all types of infections	FALSE	389 (30.3)	**783 (61.0)**	111 (8.7)
Antibiotics are strong medicines	TRUE	**1,098 (85.6)**	98 (7.6)	87 (6.8)
Antibiotics can cause side effects	TRUE	**858 (66.9)**	158 (12.3)	267 (20.8)
Antimicrobial resistance means that if you take too much antibiotics, the germs in the body get used to it and will not be killed by the antibiotics any more	TRUE	**541 (42.2)**	308 (24.0)	434 (33.8)

NB: Bold scores indicate the highest frequency of response for each item.

### 3.3 Prevalence of non-prescribed use and misuse behaviors

Self-reported misuse was common, with nearly one-quarter of participants (22.8%) acknowledging that they had obtained antibiotics without a prescription. Among these, the primary source was private/community pharmacies (81.7%), followed by family/friends (13.4%). Other behaviours of concern included the retention of leftover antibiotics for future use and the sharing of medication with others, both reported by approximately 17% of participants. Notably, more than one in four participants (26.7%) admitted to discontinuing their course of treatment early once symptoms improved, a critical driver of resistance selection.

### 3.4 Social norms and expectations

Antibiotic use was strongly influenced by social context and entitlement beliefs. As shown in Table 3, a substantial proportion of participants reported feeling pressure from their social circles or believing that non-prescribed use is a social norm. Notably, nearly 60% of participants expressed impatience with illness recovery, expecting antibiotics to *“heal them quickly”*.

**TABLE 3:** Social norms, patient demand and expectations regarding antibiotic use (N=1283).

Behavioral domain	Item description	Agreement; n (%)
Social pressure (Factor 2)	My family or friends would suggest I buy antibiotics without a prescription	286 (22.3)
	It is common in my community to use antibiotics without a prescription	287 (22.4)
Patient demand (Factor 5)	I am allowed to demand that the healthcare provider must give me antibiotics	468 (36.5)
	I expect antibiotics to heal me quickly	2 (57.9)

### 3.5 Bivariate associations with context

Pearson’s chi-square tests revealed significant associations between geographic context and social norms. Agreement with the statement *“It is common in my community to use antibiotics without a prescription”* differed significantly by residence (p < 0.001). This perception was most prevalent among participants residing in townships (36.3%) compared to those in rural areas (16.0%).

### 3.6 Determinants of misuse (multivariate analysis)

GLMs were fitted to identify independent predictors of misuse behaviors.

#### 3.6.1 Predictors of premature discontinuation

A GLM (gamma distribution) assessed predictors for discontinuing antibiotics early. The model demonstrated that sociodemographic variables were not independent predictors of this behavior. However, social norms were highly significant (p < 0.001). Participants exposed to positive social influence (where family/friends do not endorse non-prescribed use) were significantly less likely to discontinue treatment prematurely (Coeff = -0.053), highlighting the protective role of household norms.

#### 3.6.2 Predictors of non-prescribed access

The multivariable analysis identified distict predictors for different types of misuse. The independent associations between sociodemographic factors, CAMUS domains, and antibiotic misuse behaviors are detailed in Table 4. In Model 1, which predicted higher scores on the antibiotic misuse scale (e.g., premature discontinuation), health literacy and social norms were significant predictors. In Model 2, which predicted non-prescribed use, health literacy remained the strongest predictor; participants with inadequate health literacy had substantially higher odds of obtaining antibiotics without a prescription (aOR=13.86; p=0.002) compared to those with adequate literacy. Sociodemographic factors, including age (p=0.189), gender (p=0.343), and place of residence (p=0.532), were not significant independent predictors. However, behavioral determinants were highly significant.

- Social norms: Participants endorsing social norms supportive of non-prescribed antibiotic use were significantly more likely to engage in non-prescribed use (aOR=2.56; 95% CI: 1.91–3.44; p < 0.001).
- Health literacy: Participants with inadequate health literacy had significantly higher odds of obtaining antibiotics without a prescription compared to those with adequate literacy (aOR=13.86; 95% CI: 2.60–73.93; p=0.002). As detailed in Supplementary Table S1, the health literacy sub-sample differed demographically from the non-selected group (being older and more frequently employed). However, since age and employment were controlled for in the multivariable model and were not themselves significant predictors of misuse (p > 0.05), the association between health literacy and misuse represents a strong independent association distinct from socioeconomic status.
- Knowledge: Lower knowledge scores (indicating endorsement of misconceptions) were associated with a significantly increased likelihood of non-prescribed use (aOR=2.74; 95% CI: 1.84–4.09; p < 0.001).
- Patient demand: Unlike social norms, individual patient demand (Factor 5) was not a significant independent predictor in the adjusted model (p > 0.05). This distinction suggests that perceived community expectations may exert a stronger influence on behaviour than individual entitlement alone.

**TABLE 4:** Multivariable generalized linear models predicting antibiotic misuse behaviours.

Predictor	Model 1: Premature discontinuation		Model 2: Non-prescribed use	
	Coeff (B)	p-value	aOR (95% CI)	p-value
Sociodemographics				
Age	—	0.060	—	0.189
Sex	—	0.191	—	0.343
Education	—	0.998	—	0.946
Residence	—	0.712	—	0.532
Health literacy				
Inadequate (vs Adequate)	−0.337	<0.001	**13.86 (2.60–73.93)**	**0.002**
CAMUS domains				
Factor 1: Knowledge	−0.183	<0.001	**2.74 (1.84–4.09)**	**<0.001**
Factor 2: Social norms	**−0.205**	**<0.001**	**2.56 (1.91–3.44)**	**<0.001**
Factor 5: Patient demand	0.047	0.157	**0.75 (0.47–1.20)**	**0.235**

NS., ns=not significant (p > 0.05); Coefficients (B) are reported for Model 1 (premature discontinuation), while adjusted odds ratios (aOR) are reported for Model 2 (non-prescribed use). Bold scores indicate the highest frequency of response for each item.

## 4 Discussion

As the most extensive behavioral survey on antimicrobial use within the South African ambulatory care public sector to date (n=1,283), this study moves beyond descriptive surveillance to identify specific factors associated with antibiotic misuse in primary care in the country. By utilizing the psychometrically validated CAMUS instrument, the findings challenge the traditional assumption that misuse is driven solely by knowledge deficits, revealing instead that social norms and health literacy gaps are key contributors to inappropriate access.

### 4.1 The knowledge-behaviour gap

While general awareness of antibiotics was relatively high, misconceptions about viral infections remained widespread in this study (76.5%). This confirms that antibiotic misconceptions in South Africa transcend socioeconomic status, as these rates are virtually identical to those recently reported among private sector patients (Balliram and Suleman, 2025). However, recent studies by Maluleke et al. (2025a), Maluleke et al. (2025b), Maluleke et al. (2026a) in a rural South African province found low rates of non-prescribed antibiotic dispensing for viral infections in community pharmacies, with pharmacists typically offering symptomatic relief instead (Maluleke et al., 2025b; Maluleke et al., 2025c; Maluleke et al., 2026a). This stands in contrast to the extensive prescribing of antibiotics for self-limiting infections often observed within public sector PHC facilities (Lagarde and Blaauw, 2023; Chigome et al., 2025a; Maluleke et al., 2026a). This potential overuse at the clinic level may inadvertently reinforce patient misconceptions that antibiotics are necessary for flu-like symptoms. Furthermore, previous studies suggest that suboptimal knowledge regarding AMR among PHC nurses, similar to findings in other LMICs, combined with perceived patient pressure, can exacerbate this inappropriate prescribing (Lagarde and Blaauw, 2023; Saleem et al., 2025a).

Critically, while lower knowledge scores were statistically associated with misuse (aOR=2.74), knowledge alone did not protect against social pressure. This aligns with global critiques of the ‘information deficit model,’ which argue that social necessity often overrides factual knowledge in resource-constrained settings (Chandler et al., 2016). Consequently, educational campaigns focusing solely on “what antibiotics are” may fail. To be effective, interventions must address the social utility of medicines rather than just factual accuracy, including addressing widely held misconceptions among both patients and prescribers in PHC facilities about the effectiveness of antibiotics for self-limiting viral infections.

### 4.2 The “township effect” and social norms

We identified a distinct “township effect,” where norms favoring non-prescribed use were significantly higher in peri-urban settlements (36.3%) compared to rural areas (16.0%). This suggests that rapid urbanization and permissive social networks, rather than just geographic access, are accelerating the normalization of misuse. These social factors appear to be more influential drivers than simple geographic access. In these high-density environments, antibiotics appear to be shared and recommended within social networks as a pragmatic response to illness, overriding clinical guidelines. This phenomenon was also seen in the study of Tesema et al. where there was appreciable use of antibiotics sourced from friends or relatives for young children < 5 years of age with a fever or cough as opposed to obtaining them from PHC clinics (Tesema et al., 2025). This may also reflect patients storing antibiotics for future use when they feel better as opposed to long waiting times and costs to see HCPs in public PHC clinics (Maluleke et al., 2026a). A potential explanation for the lower rates in rural areas is the relational nature of care. Patients in rural settings often possess stronger social ties with community pharmacy personnel and may be more willing to accept professional guidance on the appropriate management of self-limiting viral infections which typically includes symptomatic relief first (Maluleke et al., 2025b; Maluleke et al., 2025c; Maluleke et al., 2026a). This contrasts with the experience of patients visiting public PHC facilities. In the public sector, antibiotics are frequently prescribed for viral conditions due to severe time constraints and the perception among nurses that antibiotics are necessary to manage patient expectations or prevent secondary infections (Lagarde and Blaauw, 2023; Chigome et al., 2023; Chigome et al., 2025a; Chigome et al., 2025b). Consequently, the clinic environment itself may inadvertently reinforce the belief that “sickness requires antibiotics”, which can lead to parents giving their young children left over antibiotics for their fever or cough, dispensed from community pharmacies for a previous infection, without consulting HCPs in public PHCs for their children (Tesema et al., 2025). However, the type of infection may also play a critical role. While Maluleke et al. found low rates of non-prescribed dispensing for respiratory infections, they observed extensive dispensing of antibiotics without a prescription for patients presenting with sexually transmitted infections (STIs) (Maluleke et al., 2026a). Patients often preferentially visited community pharmacies for STIs due to the perceived anonymity, friendly service, and a desire to avoid the stigma or “shyness” associated with public clinics (Maluleke et al., 2025a; Maluleke et al., 2026a). Since our study did not distinguish between infection types, the “reasonable rates” of non-prescribed use (22.8%) observed in our cohort may partially also reflect this specific demand for STI self-treatment, driven by privacy concerns rather than just clinical misconceptions.

### 4.3 Real-world prevalence vs simulated studies

Our data reveal a higher prevalence of non-prescribed access (22.8%) than previously estimated by “simulated client” studies and other local surveys (Anstey Watkins et al., 2019; Do et al., 2021; Mokwele et al., 2022). This discrepancy may be driven by the specific clinical indications for which patients seek antibiotics as well as using leftover antibiotics dispensed for a previous infection among family members or friends (Tesema et al., 2025; Maluleke et al., 2026a). Recent mystery shopper and other studies in South Africa have demonstrated that while community pharmacists generally adhere to stewardship guidelines for URTIs, resulting in low dispensing rates for these conditions (Mokwele et al., 2022; Maluleke et al., 2025b; Maluleke et al., 2025c), inappropriate dispensing increases significantly for suspected STIs (Maluleke et al., 2025a; Maluleke et al., 2026a). Consequently, as mentioned, the higher prevalence observed in our self-reported data likely captures this specific demand for STI treatment as well as potentially the use of left over antibiotics when treatment was stopped once patients felt better (Tesema et al., 2025; Saleem et al., 2025a). Since, as mentioned, we did not stratify non-prescribed purchasing by in indication as well as all potential sources of antibiotics in this study, future research should explicitly investigate the link between specific infectious syndromes and pharmacy-seeking behavior.

### 4.4 The critical role of health literacy

Health literacy emerged as a robust independent predictor of misuse, with inadequate literacy associated with a 13-fold increase in non-prescribed access. Although measured in a sub-sample, this association persisted after adjusting for education and employment. This persistence highlights functional health literacy as a distinct target for intervention. Furthermore, differences between assessed and non-assessed participants were driven by site allocation rather than self-selection, supporting the validity of this finding.

### 4.5 Nuancing “misuse” in community pharmacies

Community pharmacies were identified as the primary source of non-prescribed antibiotics (81.7%). While this aligns with global concerns regarding non-prescription sales, the interpretation of “misuse” in the South African context requires nuance. Unlike many other African nations where informal markets are dominant, South African patients rely heavily on formal healthcare structures for the management of their infectious diseases (Maluleke et al., 2026b). Consequently, our data likely captures a composite of factors: (1) illegal sales, (2) legal pharmacist-initiated therapy, and (3) provider-directed purchasing during clinic stock-outs, which is a phenomenon where nurses advise patients to purchase medication privately due to public sector shortages (Magadzire et al., 2015; Maluleke et al., 2025a; Maluleke et al., 2025b). Thus, high rates of “non-prescribed use” may reflect a systemic failure in public supply chains as much as behavioral non-compliance. Having said this, there are extensive ongoing measures to improve supply chains in South Africa, and there was extensive prescribing of antibiotics in rural public PHC facilities for URTIs without asking patients to purchase these from community pharmacies (Falco et al., 2023; Maluleke et al., 2025a; Maluleke et al., 2025b; Maluleke et al., 2026a). Additionally, the specific infectious condition plays a critical role. As noted, patients with suspected STIs often intentionally bypass public PHC facilities to avoid stigma and long waiting times, preferentially seeking treatment at community pharmacies (Maluleke et al., 2025a; Maluleke et al., 2026a). Disentangling these drivers, distinguishing between systemic access barriers, stigma-driven seeking behavior, and genuine misuse, will be a priority for future research to ensure that stewardship interventions in South African ambulatory care are appropriately targeted.

### 4.6 Patient entitlement and provider coping

Although “patient demand” was not a statistical predictor of non-prescribed purchasing of antibiotics in our model, the high prevalence of expectations (36.5% of patients feeling they can request antibiotics) presents a latent risk. In resource-constrained settings, these expectations likely manifest as contextual pressure on prescribers especially if affordability is also an issue with high levels of unemployment in our study. Prescribing antibiotics often becomes a “coping mechanism” for clinicians to manage high patient volumes and maintain patient satisfaction (Kotwani et al., 2010; Lagarde and Blaauw, 2023; Saleem et al., 2025a). This finding highlights that future ASPs cannot rely solely on gatekeeping. They must equip all HCPs with the necessary communication tools to manage expectations without eroding trust. This includes issues of language especially where terms such as antibiotics and AMR are not in the current native language (Mokoena et al., 2021; Sono et al., 2024b; Sono et al., 2025a; Sono et al., 2025b).

### 4.7 Addressing social norms through behavioural interventions

Since misuse in our study appears to be driven more by social norms and family pressure than by simple knowledge deficits, we believe any ASP must move beyond passive education to target the ‘household unit’. Evidence-based behavioral strategies include:

- Public commitment campaigns: Displaying “antibiotic commitment” posters in clinic waiting areas where prescribers and patients publicly pledge to preserve antibiotics. This leverages the same social pressure mechanism but redirects it toward responsible stewardship, a strategy previously shown to reduce inappropriate prescribing in clinical trials (Meeker et al., 2014).
- Reframing “care”: Public health messaging should reframe the narrative of “good parenting.” Instead of equating care with demanding strong medicines, campaigns should model the refusal of unnecessary antibiotics as a protective act (Tonkin-Crine et al., 2017).
- Community health worker (CHW) engagement: Leveraging South Africa’s extensive CHW network to conduct household-level dialogues, dismantling the “township effect” of misinformation within the home environment itself (Schneider et al., 2018).
- Encouraging community pharmacy personnel to speak with patients about the effective management of infectious diseases, and their prevention, including for self-limiting viral infections and STIs. In addition, the importance of completing the full course of antibiotics as prescribed (Maluleke et al., 2025d; Maluleke et al., 2026a; Saleem et al., 2025a).

## 5 Limitations

Several limitations should be acknowledged. Firstly, as noted in the methodology, the use of a convenience sampling approach with only seeking patients with ‘medical stability’ coupled with the lack of refusal data introduced selection bias. Consequently, this limits the generalizability of our findings to the broader South African ambulatory care population. Secondly, the cross-sectional design precludes causal inference; we can report associations but cannot confirm whether social norms cause misuse. Thirdly, while the sample was large (n=1,283) and diverse, it was restricted to public sector facilities in two provinces in the country, limiting generalizability to the private sector or other regions of South Africa. Fourthly, behavioral data were self-reported, which may introduce social desirability bias, although the use of trained field workers and confidentiality assurances aimed to mitigate this. Fifthly, a critical limitation was restricting participation to English-speaking patients, necessitated by the validation language of the CAMUS tool. We again explicitly acknowledge that this exclusion introduces selection bias, as non-English speakers may possess different health literacy levels and cultural norms regarding antibiotic use. Future research must prioritize the translation and cultural adaptation of CAMUS to ensure equitable inclusion of South Africa’s diverse linguistic groups.

In addition, this study also defined “non-prescribed use” based on patient self-reports of obtaining antibiotics without a medical doctor’s prescription. We were unable to differentiate between illegal dispensing, legal pharmacist-initiated therapy, or nurse-advised purchasing due to clinic stock-outs. As a result, the prevalence of “misuse” may include legitimate access pathways. Lastly, we did not ask specifically for the indication where antibiotics were purchased without a prescription. Recent research has shown just how crucial this information is. Future studies will employ provider-side verification and other information to disentangle these access routes. Despite these limitations, we believe our findings provide further guidance to all key stakeholders in South Africa to help improve future antibiotic use and reduce AMR.

## 6 Conclusion

This study provides the first theory-driven profile of antibiotic misuse in South African primary care, revealing that inappropriate use is sustained by a convergence of permissive social norms, health literacy gaps, and patient entitlement, rather than knowledge deficits alone. Consequently, our findings suggest that traditional stewardship models focusing solely on factual education or prescriber restrictions will be insufficient to counter the strong “social utility” of antibiotics in these communities. To be effective, South Africa’s National Action Plan on AMR must pivot toward behavioral interventions that target the household unit, reshape community norms, and equip healthcare workers, including community pharmacy personnel, with the skills to de-escalate patient expectations. Regarding the future utility of the CAMUS instrument, the immediate next step is to work on further item refinement, followed by the development of a condensed “short form” version of the tool suitable for rapid assessment in busy clinical settings. Following this refinement, the shortened instrument will undergo linguistic adaptation across South Africa’s diverse languages. This will facilitate its eventual integration into national surveillance strategies as a standardized metric to benchmark community antibiotic demand and evaluate the effectiveness of future stewardship interventions.

## Data Availability

The original contributions presented in the study are included in the article/Supplementary Material, further inquiries can be directed to the corresponding authors.
