# Supplementary material for: Factors associated with inappropriate antibiotic use in South African communities: findings from the CAMUS survey
Source: Front Pharmacol. 2026 Mar 20;17:1771309. doi: 10.3389/fphar.2026.1771309 (PMC13047338; doi:10.3389/fphar.2026.1771309)
Supplement: Supplementary file 1 [file Supplementaryfile1.pdf]

Supplementary Material

Supplementary Tables

Supplementary Table S1: Sensitivity analysis of the health literacy sub-sample

Comparison of sociodemographic characteristics between participants who completed the Health Literacy assessment (HELT-LL) and the non-selected group to assess potential selection bias.

Variable	Category	HELT-LL Group (n=463)	Non-Selected Group (n=813)	p-value
**Age Group**	20–29 Years	106 (22.9%)	346 (42.5%)	<0.001*
	30–39 Years	126 (27.1%)	191 (23.5%)	
	40–49 Years	104 (22.4%)	97 (11.9%)	
	50+ Years	127 (27.6%)	179 (22.0%)	
**Gender**	Female	246 (53.1%)	526 (64.7%)	<0.001*
	Male	217 (46.9%)	287 (35.3%)	
**Employment**	Unemployed	144 (31.1%)	451 (55.5%)	<0.001*
	Employed/Self	319 (68.9%)	362 (44.5%)	
**Parental Status**	Have Children	375 (81.0%)	637 (78.4%)	0.263
	No Children	88 (19.0%)	176 (21.6%)	

Notes: Analysis excludes participants with incomplete demographic data. *Statistically significant difference ($p<0.05$). The HELT-LL sub-sample was significantly older, had a higher proportion of males, and higher employment rates than the non-selected group. To mitigate this bias, all multivariable models were adjusted for age, sex, and employment status.

Supplementary Table S2: Model diagnostics for Generalized Linear Models (GLM)

Comparison of distribution families for the continuous outcome variable (Misuse Score) to justify the selection of the Gamma model.

Metric	Gamma Distribution	Inverse Gaussian (IGAUSS)
**Akaike Information Criterion (AIC)**	986.324	1079.932
**Bayesian Information Criterion (BIC)**	1113.828	1201.05
**Omnibus Test (Likelihood Ratio)**	p < 0.001	p < 0.001
**Conclusion**	Preferred Fit (Lower AIC)	Poor Fit

Supplementary Table S3: Spearman's Rank Correlation Matrix of CAMUS Factors

Assessing multicollinearity between behavioural determinants included in the multivariable models.

Variable	F1	F2	F3	F4	F5
**F1: Understanding of Antibiotics**	1				
**F2: Social & Behavioural Norms**	0.174**	1			
**F3: Non-prescribed Use**	-0.201**	-0.506**	1		
**F4: Understanding of AMR**	0.113**	-0.136**	0.184**	1	
**F5: Patient Demand**	0.02	-0.05	0.075**	0.226**	1

Note: ** Correlation is significant at the 0.01 level (2-tailed). All correlation coefficients (r_s) remained below the threshold of 0.7, confirming that multicollinearity was not a concern for the regression models.
